# Simplified routines in prescribing physical activity can increase the amount of prescriptions by doctors, more than economic incentives only: an observational intervention study

**DOI:** 10.1186/1756-0500-3-304

**Published:** 2010-11-15

**Authors:** Gerthi Persson, Ingvar Ovhed, Eva Ekvall Hansson

**Affiliations:** 1 Blekinge Centre of Competence, Landstingets Kansli, SE-371 81 Karlskrona, Sweden; 2 Lund University, Department of Clinical Sciences in Malmö/General Practice/Family Medicine, Malmö University Hospital SE-205 02 Malmö, Sweden

## Abstract

**Background:**

Physical inactivity is one well-known risk factor related to disease. Physical activity on prescription (PAP) has been shown in some studies to be a successful intervention for increasing physical activity among patients with a sedentary lifestyle. The method involves motivational counselling that can be time-consuming for the prescribing doctor and might be a reason why physical activity on prescription is not used more frequently. This study might show a way to make the method of prescribing physical activity more user-friendly. The purpose is to determine whether a change in procedures increases the use of physical activity on prescription, and thus the aim of this study is to describe the methodology used.

**Results:**

The observational intervention study included an intervention group consisting of one Primary Health Care (PHC) clinic and a control group consisting of six PHC clinics serving 149,400 inhabitants in the County of Blekinge, Sweden.

An economic incentive was introduced in both groups when prescribing physical activity on prescription. In the intervention group, a change was made to the process of prescribing physical activity, together with information and guidance to the personnel working at the clinics. Physical therapists were used in the process of carrying out the prescription, conducting the motivational interview and counselling the patient. This methodology was used to minimise the workload of the physician. The chi-2 test was used for studying differences between the two groups. PAP prescribed by doctors increased eightfold in the intervention group compared to the control group. The greatest increase of PAP was seen among physicians in the intervention group as compared to all other professionals in the control group. The economic incentive gave a significant but smaller increase of PAP by doctors.

**Conclusion:**

By simplifying and developing PAP, this study has shown a concrete way to increase the implementation of physical activity on prescription in general practice, as opposed to what can be gained by an economic bonus system alone. This study indicates that a bonus system may not be enough to implement an evidence-based method.

## Background

Based on current knowledge and understanding of the relationship between physical activity and health, it is important for the health care system to provide information regarding methods of treating and preventing illness through physical activity [1].

Physical inactivity is estimated to cause 6% of the burden of disease for men and 3% for women in Sweden [2].The standard treatment for illnesses caused by decreased physical activity and an unhealthy diet is drugs, hence the cost of drugs is high. Drugs to reduce blood pressure, blood lipids and blood sugar account for 15% of the total amount of Swedish drug costs [3]. WHO has estimated that 80% of heart and cardiovascular disease, 90% of non-insulin-dependent diabetes and 30% of all cancer can be prevented merely by a change of life-style. This would include altering bad eating habits, quitting smoking and participating in the desired amount of physical activity [3].

As a primary care provider it is essential to influence patients to become more aware that lifestyle changes will make a difference in reducing lifestyle-related diseases. By using Motivational Interviewing (MI) the care provider recognises the fact that patients who need to make lifestyle changes shows different levels of readiness for a behaviour change [4]. In addition to using MI, the doctor can prescribe physical activity. Prescribing physical activity, in Sweden referred to as PAP (Physical Activity on Prescription), has been shown to be one method to enhance the importance of physical activity as a treatment for a number of diagnoses [5]. Several ongoing studies will determine the effectiveness of PAP [5-7]. The book "FYSS" describes evidence for physical activity on a primary as well as a secondary level of prevention. Physical activity may be used as a complement, or even a substitute, for drugs treating several diagnoses [8]. Compliance with PAP is 56% according to a non-randomised study performed in Östergötland, Sweden [9]. Prescriptions of drugs show a compliance of 50% [9] Studies demonstrate that PAP and doctors referrals to an exercise specialist are two cost-effective methods among many healthcare-based interventions aimed at promoting physical activity in primary health care [10,11]. According to the Surgeon General, 30 minutes of daily exercise is recommended [12]. A Swedish national survey of physical activity revealed that 46% of women and 42% of men aged 18 to 84 were physically active with moderate intensity less than 30 minutes a day [13]. Determinants for level of physical activity are: age, level of education, ethnic origin, urban or rural living and physically inactive friends [14].

There appears to be sufficient evidence for PAP as an effective method for increasing physical activity [5,6,9,10,15,16]. However, since it also appears that PAP is insufficiently used in Primary Health Care (PHC) [8,15,16] it seems important to elucidate methods for promoting the use of PAP in PHC. The aim of this study was to ascertain whether involving a physical therapist to perform the motivational interview and to determine duration, intensity and activity could increase the amount of PAP, compared to an economic incentive only.

## Methods

### Material

This is an observational intervention study of pilot character conducted from 2006 to 2007. The County of Blekinge is divided into eight PHC clinics serving 149,400 inhabitants. The intervention group consisted of one PHC clinic with 11 family physicians serving 19,877 inhabitants in one town with 49.0% women and 7.8% born outside of Sweden. The control group was composed of six PHC clinics with 46 family physicians serving 80,592 inhabitants, 49% living in the country, 49.0% women, 8.7% non- Swedish- born residents. One of the clinics was excluded from the study because they had been using PAP prior to the intervention. This study was made possible when the PHC clinic forming the intervention group decided to change routines for prescribing physical activity. The intervention clinic is also the largest unit in this area and was therefore selected to become the intervention group. The remaining PHC clinics in the region were selected as a control group. The foreign population showed a high incidence of lifestyle-related symptoms and diseases, but unpublished data from another study show that non-Swedish-born residents did not receive a high rate of PAP. When needed, an interpreter was used in those cases where a prescription was written.

### Intervention

The intervention consisted of a doctor initiating PAP. The prescription was sent electronically to a physical therapist. The patient was scheduled for a motivational interview within a couple of days. During the appointment the patient decided, with guidance from the physical therapist, what type of physical activity to perform in order to influence his or her condition. Besides the type of activity to perform, the duration and intensity were prescribed. All personnel in the intervention group received quarterly information regarding numbers of prescribed PAP.

The control group continued to prescribe PAP using existing referral routines involving the doctor to do the counselling of the patient without support from the PT. The intervention and the control group used the same prescription form.

Concurrently with the start of the study, both groups were introduced to a bonus system for prescribing PAP. A target for prescribed PAP was connected to the number of patients listed per clinic. The target included prescriptions from all personnel including nurses and physical therapists. This study will only look at PAP prescribed by doctors. If the target was met, the clinic was awarded a sum ranging from SEK 7,700 ($1,000) to SEK 25,900 ($3,400), depending on the size of the clinic. Thus a first level of intervention was common to both groups. This study measured the second level of intervention. The second level was a change in routines for the doctor when prescribing physical activity. The doctors were able to receive assistance with the consultation regarding the type, duration and intensity of activity.

### Statistics

A chi-2 test was used to measure the differences in number of PAP in 2006 compared to 2007 within the groups and differences between the two groups in 2006 and 2007. Statistica version 9 was used for statistical analyses.

### Ethics

The study was made possible as an improvement project at the PHC clinic. All data concerning PAP activity were collected from computerised medical journals with the approval of the directors of the clinics no individuals where identified in the data. The number of PAP prescriptions was counted and no other information concerning patient data was made accessible. The prescribing physician, nurse and physical therapist were anonymous.

## Results

Table 1 shows the differences between the two groups according to the proportion of PAP prescriptions and the difference between the two groups according to the change of prescribed PAP between 2006 and 2007. The increase of prescriptions in the intervention group was significant (p = 0.0000). The increase in the control group was less and not significant (p = 0.0751). In 2006, the intervention group prescribed PAP 8 times during a total of 19.035 consultations. This was not significantly different from the control group prescribing PAP 58 times during a total of 84.554 consultations (p = 0.1894). However, in 2007 a significant difference was seen between the two groups (p < 0.000). In the intervention group, a change was seen from 4.20 PAP in 2006 to 33.43 PAP/10,000 consultations in 2007, whereas the control group showed an increase from 6.86 PAP in 2006 to 9.30 PAP/10,000 consultations in 2007. Five out of seven PHC clinics reached the target for receiving the financial reward, indicating that the target was rather modest.

**Table 1:** Differences in number of PAP 2006 compared to 2007 within the groups and differences between the two groups in 2006 and 2007.

	Intervention group	Control group	
	**PAP/total numbers of consultations**	**PAP/total numbers of consultations**	**p-value**

2006	8/19.035	58/84.554	< 0.1894
2007	68/20.339	82/88.175	< 0.0000
p-value	< 0.0011	< 0.0751	

# Differences in numbers tested by chi-2 test

## Discussion

Analysis of this intervention study over 2 years showed an increase of PAP prescribed by physicians when a change of routine was made. The purpose of the change was to minimise the workload for doctors when using PAP as a treatment for lifestyle-related conditions. By involving a physical therapist to do the motivational interview and to suggest activity, duration and intensity, the likelihood of using PAP as a treatment increased. The intervention made a favourable impact on the number of PAP prescriptions and a significant increase of prescriptions was seen in the intervention group. An increase of prescriptions was also seen in the control area, but this was not significant.

A bonus system connected to money was introduced in the intervention and control area concurrently. It may be expected that this bonus system has had a similar impact on prescription rate in all participating areas. The authors have unfortunately not been able to control for any potential confounding factors other than the economic incentive, a limitation in the study. In addition to the introduction of the bonus system, PAP was made accessible by computer, which made it easy to use for both the intervention group and the control group prior to the intervention. This study did not measure compliance with PAP, or the long-term effect of prescribing physical activity, since the only aim was to increase the numbers of PAP prescriptions by doctors. It may be seen as an attempt to stimulate the use of PAP at PHC clinics in the County of Blekinge. As seen in this study, it is important for health professionals to work together when influencing the patient to change lifestyle [17]. We were unable to randomise the population in the study, and thus the results should be viewed with caution.

As in this study, other studies also show an increase of prescriptions after small modifications to the method along with computerised development [9]. Nevertheless, there was a significantly better result for PAP prescribed by doctors in the intervention group.

The PAP process as described made it less time-consuming [18] for the doctors and facilitated the use of the method, although more time was consumed together with the PT doing the motivational interview. A part of the population in both groups consisted of non-Swedish-born residents. Unpublished data show that very few of the non-Swedes have received PAP and when it occurred an interpreter was used. We believe that it has not affected the results of the study. By using a team approach, lifestyle change is emphasised [1-3,8,18]. As seen in this study, it is important for health professionals to work together when influencing the patient to change lifestyle [17]. Other clinicians such as the nurse play an important role when it comes to counselling the patient to a lifestyle change [16]. Physicians and nurses in counselling sessions are likely not to comment on or react to patients' statements regarding factors that encourage or discourage their use of physical activity [19]. It has yet to be shown which health professional makes the greatest impact on patients' change of lifestyle when it comes to counselling patients on appropriate physical activity. We believe the message's impact concerning lifestyle change becomes much greater if both the family physician and the physical therapist are involved when prescribing, similar to previous studies [18]. When promoting a physically active lifestyle, there are two points of particular importance. First, the patient should be informed of his/her health and any feasible methods of treatment. Second, the patient should, when appropriate, be given information on methods to prevent injury or illness [13]. To use PAP as a treatment along with counselling the patient to an increasingly less sedentary lifestyle might in the short term need more time than prescribing a drug for a life-style-related disease-time the doctor lacks in the clinic, whereas for the physical therapist the main assignment is to promote physical activity. We believe the message's impact concerning lifestyle change becomes much greater if both the family physician and the physical therapist are involved when prescribing, similar to previous studies [18]. When promoting a healthy lifestyle, it is necessary for the physician to support the message of becoming more physically active [17].

Confusion prevails regarding the nomenclature when prescribing physical activity, as some studies refer to Exercise on Prescription, EoP [6,7] whereas others refer to Physical Activity on Prescription, PAP [5,15], or Physical Activity Referral, PAR [16], and we hope that the choice of words can be defined since the context and meaning seem to vary in the studies.

According to the Swedish Institute of National Health, preventive efforts account for only 5% of Swedish health care costs [10,13]. Among other methods, prescribing physical activity is an evidence-based method in primary and secondary prevention, yet few patients are treated with PAP, according to findings that this study also indicates [20,21].

Data from our study included 8 out of 9 PHC centres in the County of Blekinge, a small population but a homogeneous intervention and control group in a geographically defined area. Future randomised controlled studies involving many PHC clinics as an intervention group might give more knowledge about how to increase the frequency of PAP.

Many factors are likely to influence why doctors choose or choose not to use PAP as a treatment, but still primary care must continue to promote appropriate referral pathways [22].A systematic review finds PAP acceptable and feasible to doctors, and most patients are willing to receive a prescription [16]. How will the health care system responds to a sedentary patient? A team approach with a prescription written by the doctor coupled with a physical therapist doing a motivational interview may be one important feature when trying to reinforce the message of a necessary change of lifestyle. Whether or not this change of referral pathway will result in a lifestyle change remains to be proved. There is a lack of knowledge regarding what type of patients can successfully be encouraged to adopt a less sedentary lifestyle. Methods need to be developed to identify patients who will gain the most from preventive counselling.

In summary, a change to an alternative pathway of prescribing physical activity will stimulate the doctor to prescribe PAP. The pathway described made it less time-consuming [18] for the doctor and facilitated the use of the method, although more time was consumed together with the PT doing the motivational interview. This observational study merely describes a way to increase prescriptions of PAP.

## Conclusion

By simplifying and developing PAP, this study has shown a concrete way to increase the implementation of physical activity on prescription in general practice, as opposed to what can be gained by an economic bonus system alone. This study indicates that a bonus system may not be enough to implement an evidence-based method.

## Competing interests

The authors declare that they have no competing interests.

## Authors' contributions

GP participated in the design of the study, performed the statistical analysis and drafted the manuscript. IO participated in the design of the study and helped to draft the manuscript. EEH helped to draft the manuscript. All authors read and approved the final manuscript.
